# Intestinal Paracoccidioidomycosis: Case report and literature review

**DOI:** 10.1016/j.ijscr.2022.106801

**Published:** 2022-01-29

**Authors:** Felipe Giron, Marco Vanegas, Lina M. Rodriguez, Valentina Hernandez-Santamaria, Carlos Eduardo Rey Chaves, Jairo Ortega

**Affiliations:** a Faculty of Medicine, Universidad del Rosario, Colombia; b Faculty of Medicine, Universidad de los Andes, Bogotá, Colombia; c Hospital Universitario Mayor de Mederi, Colombia

**Keywords:** Intestinal Paracoccidioidomycosis, General surgery, Case report

## Abstract

**Introduction and importance:**

Paracoccidioidomycosis (PCM) is a systemic fungal infection, primarily affecting the respiratory tract. Extra pulmonary presentation is rare, representing less than 1% of cases (about 1 in every 200 cases).

**Case presentation:**

We present a case of a 73-year-old male with acute surgical abdomen secondary to Intestinal Paracoccidioidomycosis requiring intestinal resection and postoperative antifungal therapy.

**Conclusion:**

Intestinal Paracoccidioidomycosis represents a rare pathology with challenging diagnostic approach due to its frequency and nonspecific clinical manifestations. Extra pulmonary presentation is rare, but it should be considered in endemic regions.

## Highlights

• Extrapulmonary presentation of PC it's a rare entity, reaching just 1% of the cases.
• Fungal infection should be in the knowledge of general surgeon
• IP, represents a surgical challenge, a multidisciplinary approach needs to be done in order to achieve good outcomes.

## 1 Introduction and importance

Paracoccidioidomycosis (PCM) is a systemic fungal infection, primarily affecting the respiratory tract. Extra pulmonary presentation is rare, representing less than 1% of cases (about 1 in every 200 cases) [1], being more frequent in patients with risk factors that favor hematogenous dissemination, among which the male gender generally stands out in the context of immunosuppression. We report one such case of intestinal PCM, managed in a fourth level public hospital. Work has been reported in accordance to SCARE guidelines [2].

## 2 Case presentation

After ethical and institutional approval, previous informed consent filled, following SCARE guidelines [2]. A 73-year-old male presented with migratory acute abdominal pain (4/10 Visual Analogue Scale (VAS)) to the Emergency Department. He stated that it started in the epigastrium and then it was referred to the right lower quadrant and lumbar area. Patient medical history included chronic obstructive lung disease, hypertension, dissection of the common iliac artery of 22 mm, with 27% stenosis of the superior mesenteric artery reported in a computed tomography angiogram (CT Angiogram) (2020), lumbar cyst resection, documented ulcer in the hepatic angle of the colon, (negative for inflammatory intestinal disease and malignancy, 2018), and chronic lumbar pain.

Physical examination was performed finding a painful abdomen with voluntary muscular defense with greater intensity on the lower quadrants. He had no fever or dyspnea. White-cell count was elevated with neutrophilia (14.870 per microliter, 77.2% neutrophils). Other checkups reported a non-inflammatory urinalysis, negative urine gram stain, arterial gases with compensated metabolic acidosis, without hyperlactatemia and a positive fecal occult blood test.

**Fig. 1:**
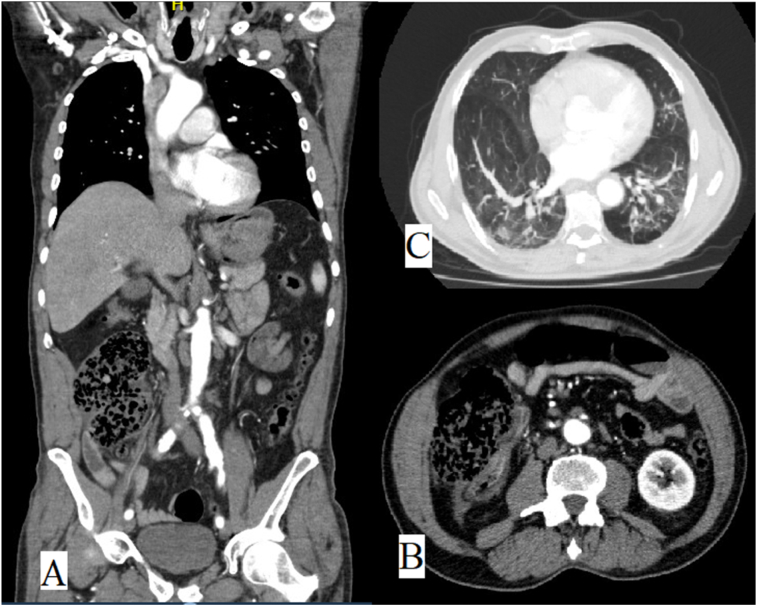
A. Tomographic findings in ascending colon. B. Tomographic findings in ascending colon (sagital view). C. Tomographic findings in pulmonary tissue.

Due to his medical history, a CT angiogram was performed disclaiming vascular pathology, no evidence of nephrolithiasis, urolithiasis, and appendicitis. Diverticular disease of the descending and sigmoid colon without signs of complication was reported, no signs of pneumoperitoneum, nevertheless inflammatory process of ascending colon associated to small bowel and ascending colon dilation with alteration of adjacent fat was documented (Fig. 1). Despite adequate broad spectrum empiric antibiotic regimen, analgesic and optimal intravenous fluid resuscitation, the patient's general condition worsened. Patient abdominal pain increased 10/10 VAS, with peritoneal irritation at physical examination, therefore, patient was transferred to the operating room (OR) to perform an exploratory laparotomy.

A right hemicolectomy with segmental jejunum resection, transverse ileum, and jejunum jejunal anastomosis were performed by an experienced general surgeon. Intraoperative findings showed a solid intraluminal lesion in the cecum of 7 cm (greater diameter), cecum walls of necrotic appearance, without evidence of perforation or free liquid in the peritoneal cavity. Additionally, a jejunal lesion comprising 80% of the small bowel's diameter was found at 110 cm from the Treitz ligament. and four lesions of 0,5 cm were found in the liver.

Intensive Care Unit (ICU) was required during postoperative due to patients' need of vasopressor and mechanical ventilation. Once extubated and with no vasoactive support needed, the patient was transferred to an inpatient postoperative floor, in the next days developed dysphagia, abundant production through nasogastric tube, flats present but no feces, for which parenteral nutrition and postoperative ileus management was started. Despite adequate supportive care, there was no improvement, with increased drainage through nasogastric tube, pain, and distention. Revision of abdominal cavity was performed 8 days after first intervention finding 400 cc of hemoperitoneum and both anastomoses unscathed, due to impossibility of closure of abdominal wall patient required an additional procedure 4 days later to abdominal wall closure.

Pathology reported ulcerated colonic and jejunal mucosa with presence of granulomas some of them with central necrosis, chronic inflammation that compromises subserosa immunohistochemistry reported Gomory + (Presence of fungal structures), Ziehl Neelsen - AE1/AE3 and CAM5.2 positive in cryptic epithelium confirming diagnosis of gastrointestinal Paracoccidioidomycosis.

During hospitalization patient presented nosocomial pneumonia and needed percutaneous drainage of intra-abdominal collection both treated successfully with no further complications. After 56 days of inpatient care, he was discharged with antifungal extended therapy. His controls have been uneventful up to 6 months.

## 3 Discussion

Paracoccidioidomycosis (PCM) is a systemic fungal infection generally self-limited to lung tissue caused by the dimorphic fungi *Paracoccidiodes brasiliensis*. It was first described as a disseminated disease in Buenos Aires, Argentina in 1892 [1]. Is most common in Central and South America, around 85% of cases have been reported in Brazil, followed by Colombia, Venezuela, and Ecuador [1]. In Colombia infections have been reported mainly in the departments of Guajira and Magdalena [3] hence the importance of presenting a novel case in the Andenian region of the country. *Paracoccidioides* grows in the soil of humid regions with a medium to high rainy season, mild temperatures, and the presence of forests and rivers [1], [4].

Infection is acquired by arthroconidia (infectious form of the fungus product of hyphal fragmentation) inhalation. Once the fungus invades the lungs in the yeast form, a non-specific inflammatory response is initiated and over 80% of cases are asymptomatic [1]. When symptomatic, usually present with fever, cough, dyspnea, chest pain or nonspecific and self-limited catarrhal symptoms 7 to 21 days after exposure in 39% of cases [1]. About 12% of patients with lung disease develop cutaneous manifestations that range from ulcers to verrucous lesions, which should suggest Paracoccidioidomycosis in endemic areas [5]. After full anamnesis was performed, there were no respiratory symptoms referred by the patient presented in this case.

The extra pulmonary presentation is rare, representing less than 1% of cases (about 1 in every 200 cases) [1], [6] and is associated with significant morbidity and mortality [6]. Its presentation is more frequent in patients with risk factors that favor hematogenous dissemination, among which the male gender generally stands out in the context of immunosuppression. Taking into account that the most important role in protection is played by macrophages, which are only effective if the helper lymphocytes (CD4 +) are competent [7], pregnant women, patients with active neoplastic processes and those with immunosuppression states (acquired immunodeficiency syndrome, lymphoma or transplant recipients especially) are more susceptible to presenting disseminated disease [1], [8]. To date no immunological derangements have been documented in our patient.

Gastrointestinal presentation is also infrequent, being the meninges, skin, bone, joints, mediastinum, and lymph nodes the most frequent involved organs [1], [9]. Even though Paracoccidioidomycosis is more prevalent in the lung and skin [5], it can spread by hematogenous dissemination involving different abdominal organs [10], [11], [12], causing lymphadenopathy, hepatomegaly, splenomegaly and in some cases, it can even reach the gallbladder and the retroperitoneal musculature [13]. Clinical spectrum in abdominal cases varies from abdominal pain and diarrhea to secondary appendicitis, colitis and colonic ulcers [14], [15].

There have been around 10 cases of abdominal Paracoccidioidomycosis reported in literature and given its extremely rare presentation, only clinical suspicion in endemic areas favors diagnosis [6]. As reported in this case, during initial management and until a definite histopathological report was obtained, no clinical suspicion of fungal infection was considered and thus as in most reported cases, diagnosis was an incidental finding [9]. Suspecting and diagnosing Paracoccidioidomycosis remains a challenge for science. Diagnostic tools include serological methods or histopathological and microbiological studies of the affected tissue or organs. The best diagnostic method is through a tissue biopsy or resection for histopathological study and/or culture [1]. Cytokeratin monoclonal antibodies AE1/AE3 [16] aid in its differentiation from a cancerous process, however more research is needed in this regard. The presence of spherules with endospores in tissue, bronchoalveolar lavage, sputum or other fluids is pathognomonic for infection by Paracoccidioidomycosis [6]. Serological tests have diagnostic and prognostic value for the management of the infection; however, it should be taken into account that immunocompromised patients present low rates of seropositivity [17]. The paracoccidioidin test consists of intradermal injection of standardized micelle coccidioidin, it is interpreted as positive when there is a zone of induration greater than 5 mm, is very specific and cross reactions are rare. Imaging manifestations of PCM are nonspecific, pneumonic infiltrates and mediastinal lymphadenopathy can be seen [18].

Treatment includes monotherapy of amphotericin B, ketoconazole, fluconazole, or itraconazole [19]. Although in cases of systemic disease treatments with amphotericin B associated with an azole have been described there is no evidence that supports its use [20]. Drugs such as caspofungin, posaconazole and voriconazole have been postulated as possible new agents for the management of cases of refractory infections; the first two showing effectiveness in vitro and the last showing effectiveness in some patients [19]. A series of 15 patients with amphotericin B resistant coccidioidomycosis were treated with posaconazole (800 mg/day) with an overall response of 73% [21].

PCM is a disease with high morbidity but relatively low mortality, relapses are common so it's important to receive and complete appropriate antifungal therapy [1]. Follow-up must be done monthly during the first three months of treatment and every three months thereafter, until the end of the first year [1].

## 4 Conclusion

Intestinal Paracoccidioidomycosis represents a rare pathology with challenging diagnostic approach due to its frequency and nonspecific clinical manifestations. Extra pulmonary presentation is rare, but it should be considered in endemic regions. Therefore, all cases should be published or added to scientific repositories to make data available regarding the management and outcomes for future reference and strengthening of knowledge.

## Provenance and peer review

Not commissioned, externally peer-reviewed.

## Consent

Written informed consent was obtained from the patient for publi-cation of this case report and accompanying images. A copy of the written consent is available for review by the Editor-in-Chief of this journal on request.

## Ethical approval

Ethical approval of institutional committee was made previous publication.

## Funding information

This research did not receive any specific grant from funding agencies in the public, commercial, or not-for-profit sectors.

## Author contribution

**Felipe Giron**, **MD**, **MSc**: Make substantial contributions to conception and design, acquisition of data, analysis and interpretation of data.

**Marco Vanegas**, **MD**: Participate in drafting the article and revising critically for important intellectual content.

**Lina Marcela Rodriguez**, **MD**: Participate in drafting the article and revising it critically for important intellectual content.

**Valentina Hernandez**, **MD student**: Participate in drafting the article and revising it critically for important intellectual content.

**Carlos Eduardo Rey Chaves**, **MD**: Make substantial contributions to conception and design, acquisition of data, analysis and interpretation of data.

**Jairo Ortega**, **MD**: Give final approval of the version to be submitted and any revised version.

## Guarantor

Felipe Giron.

## Research registration number

None.

## Declaration of competing interest

Authors do not declare any conflict of interest.
